# Challenges of machine learning model validation using correlated behaviour data: Evaluation of cross-validation strategies and accuracy measures

**DOI:** 10.1371/journal.pone.0236092

**Published:** 2020-07-20

**Authors:** Bence Ferdinandy, Linda Gerencsér, Luca Corrieri, Paula Perez, Dóra Újváry, Gábor Csizmadia, Ádám Miklósi

**Affiliations:** 1 MTA-ELTE Comparative Ethology Research Group, Budapest, Hungary; 2 Department of Ethology, Eötvös Loránd University, Budapest, Hungary; 3 MTA-ELTE ‘Lendület’ Neuroethology of Communication Research Group, Budapest, Hungary; University of Pittsburgh, UNITED STATES

## Abstract

Automated monitoring of the movements and behaviour of animals is a valuable research tool. Recently, machine learning tools were applied to many species to classify units of behaviour. For the monitoring of wild species, collecting enough data for training models might be problematic, thus we examine how machine learning models trained on one species can be applied to another closely related species with similar behavioural conformation. We contrast two ways to calculate accuracies, termed here as overall and threshold accuracy, because the field has yet to define solid standards for reporting and measuring classification performances. We measure 21 dogs and 7 wolves, and find that overall accuracies are between 51 and 60% for classifying 8 behaviours (lay, sit, stand, walk, trot, run, eat, drink) when training and testing data are from the same species and between 41 and 51% when training and testing is cross-species. We show that using data from dogs to predict the behaviour of wolves is feasible. We also show that optimising the model for overall accuracy leads to similar overall and threshold accuracies, while optimizing for threshold accuracy leads to threshold accuracies well above 80%, but yielding very low overall accuracies, often below the chance level. Moreover, we show that the most common method for dividing the data between training and testing data (random selection of test data) overestimates the accuracy of models when applied to data of new specimens. Consequently, we argue that for the most common goals of animal behaviour recognition overall accuracy should be the preferred metric. Considering, that often the goal is to collect movement data without other methods of observation, we argue that training data and testing data should be divided by individual and not randomly.

## Introduction

Measuring behaviour is a crucial methodological issue of behavioural studies. Since the body movements of an individual are among the main measurable components of behaviour, the usage of animal-borne motion sensor devices, such as accelerometers and/or gyroscopes, has been exponentially increasing both in the study of wild, free ranging animals from birds to fishes [1–8] and domestic animals (e.g. laboratory, farm and companion animals) [9–13]. Other types of animal-borne sensors instead measure the geographical position of animals (e.g. [14, 15]) and sometimes these are used together so both geographical position and body motion is available [16]. Such techniques are not restricted to animals and similar studies are carried out on humans as well (e.g. [17–19]), although typically it is easier to acquire human data, due to more cooperative subjects.

Main advantages of collecting motion data by means of small, lightweight sensor devices are for example the possibility to obtain measurements from undisturbed wild animals, and to access information that is unattainable—or far more complicated to obtain—with more classical observational methods, like daily rhythm, degree of activity, video recording etc. [20, 21]. Moreover, sensor-aided methods supply scientists with huge amounts of objective and quantitative data, which can not only be used for understanding the behaviour of specific species, but can be used to underpin theoretical works of animal behaviour (e.g. [22–24]).

One of the techniques used for analysing such data is applying machine learning algorithms to train models that can automatically identify distinct, pre-determined behavioural categories [2, 5, 6, 25–28], by classification of observed motion data. This can decrease the need for direct visual observation by skilled human investigators, possibly reducing the amount of infrastructure and time needed to observe animal behaviour. Although more conventional statistical methods can also lead to results (e.g. [29]), in the typical use-case scenario, the ethologist would use these methods to obtain behavioural sequences for further (ethological) analysis, and is less concerned in how the method applied extracts behaviour from the raw data. In this case machine learning is believed to be easier to use and to lead to better results [30].

Training effective cross-species models could help with observing species, where collection of data from one species is much more complicated than the other species and the two species have similar behavioural conformation. For example, models could be trained on domestic counterparts of wild animals, like dogs and wolves, or domestic cats and other small felines. Some work has already been carried out on this subject for categorizing movement behaviours [31], but the problem is far from solved in general.

In a machine learning process the gathered data needs to be labelled to establish the ground truth (typically done by a human expert), then some part of the labelled data (training set) is used to train a (machine learning) model and finally the performance of this model is assessed using some metric measured on some other part of the labelled data (the test set) [32].

Choosing how to separate the collected and labelled data into training and tests sets, and choosing a metric to measure performance of the model are in general non-trivial problems. Different types of data and different goals in mind might mean that for different problems different separation techniques and different metrics might be optimal. The data used for animal behaviour classification usually comes from very few animals (often less then 10, sometimes even one, and the largest sample of subjects consisted of 40 individuals [27, 33, 34]), while each animal contributes hundreds or thousands of data points. This means, that when randomly selecting data for testing, these points will very likely not be independent from a large part of the training set. Also, in many cases the intended use of the trained model is to apply it on the data of a new set of individuals, where labelling is not available [35, 36].

Our aim with this study is to expose two methodological issues existing in current practices related to measuring the performance of models trained to classify animal behaviour and to investigate whether data collected from family dogs can be used to categorize behaviour of wolves. We explore two ways to separate data into training and test sets, and assess two ways to measure accuracy of trained models, to reveal best practices for the analysis of behavioural data. Using our data collected from three groups of domestics dogs (*Canis familiaris*) and wolves (*Canis lupus*) we show the differences between the various methods, and make suggestions which methods provide better results for animal behaviour classification. We also show that—with more careful planning of data collection —, not just behaviours of locomotion, but feeding behaviours could be identified using cross-species models, if the two species are sufficiently similar to each other with regard to their anatomy and general movement pattern.

## Materials and methods

### Ethics statement

The currently operating Hungarian law “1998. évi XXVIII. Törvény”—the Animal Protection Act—defines experiments on animals in the 9th point of its 3^rd^ paragraph (3. §/9.). Non-invasive studies on animals are currently allowed without any special permission by the University Institutional Animal Care and Use Committee (UIACUC, Eötvös Loránd University, Hungary). We also obtained a written statement (Nr.: PEI/001/1058-4/2015) by the Food Chain Safety and Animal Health Directorate Government Office based on the decision of the Scientific Ethic Council of Animal Experiments. According to this statement and the corresponding definition by law, our non-invasive observational study is not considered as an animal experiment. Owners with their dogs responding to our advertisement on Family Dog Project’s Facebook page https://www.facebook.com/FamilyDogProject volunteered to participate, as well as the private owner of the wolves. The owners gave their written consent.

### Subjects

Our subjects were 21 healthy adult family dogs (14 purebreds from 12 different breeds and 7 mixed breeds, of which 12 males and 9 females with mean age: 5.9 years, sd = 3.3) and 7 healthy adult grey wolves (2 females and 5 males, mean age: 4.6 years, sd = 2.8). The family dogs were brought by their respective owners to the university premises from their homes, which were in Budapest, Hungary, or within close proximity of Budapest. The privately owned wolves were all hand-raised, tame animals kept with permission in a wolf park in Gödöllő, Hungary. All individuals went through a veterinary check-up prior to the experiment and were found to be in good body condition, free of any orthopaedic and neurological disorders. The dogs were split into three groups of seven individuals based on their weight, which was indicated by their owners (‘small’ sized dogs: <10 kg, mean weight: 7.6 kg; ‘medium’ sized dogs: 10-25 kg, mean weight: 16.8 kg; ‘large’ sized dogs: >25 kg, mean weight: 32.5 kg; based on weight, the wolves fall into the ‘large’ category, mean weight: 31 kg). See S1 Table for further details.

### Devices

**Fig 1:**
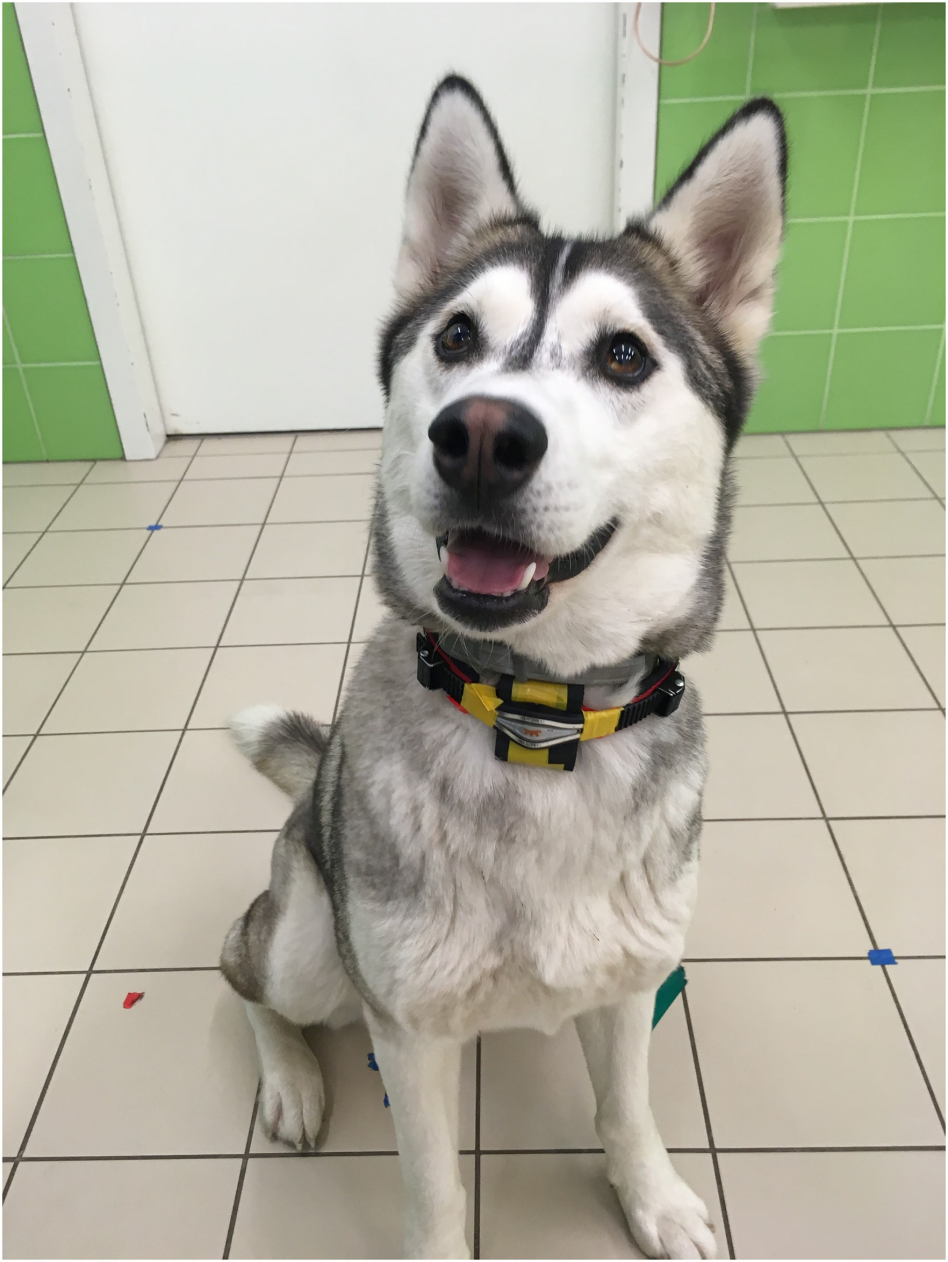
The custom made collar worn by a dog, with the inertial data logger installed.

Inertial data was gathered by commercially available Apple Watch Series 1 device (further referred to as inertial data logger), which was placed in a custom made collar, in such a manner that the device was positioned in the middle of the lower part of the animals’ neck (see Fig 1). The inertial data logger has a tri-axial accelerometer and a tri-axial gyroscope operating at 50 Hz sampling rate. The operating system running on the inertial data logger provides an API for accessing processed data, which separates gravity from the acceleration caused by the motion of the device and provides attitude (three dimensional orientation) data.

Video recordings were created with a commercially available Apple iPhone SE phone. By design of the manufacturer, the inertial data logger had to be “paired” with the phone. This allowed control of data logging via the phone and also automatic synchronization of the video recording to the inertial data collected.

### Data collection protocol

Data was collected between August and October 2016 in Budapest and Gödöllő, Hungary. Data from the dogs was collected either on open flat ground outdoors or indoors, and wolf was collected on outdoor open flat ground (i.e. their own territory). The data procedure was as follows.

1. Preparation: The animals were equipped with the collar housing the inertial data logger in such a way that it was comfortable for the animal and did not hinder any movement. Data logging was initiated on the inertial data logger and the video recording was started simultaneously.
2. Behaviour recording: The handlers were asked to instruct or coax the animals into performing the following eight, non-overlapping behaviours: sit, lay, stand, walk, trot, run, eat and drink (see S2 Table for definitions of the behaviour categories).

### Supervised learning

#### Data preparation

Following the method described in [25] videos were tagged with eight behaviour categories of interest (sit, lay, stand, walk, trot, run, eat and drink) with Solomon Coder (© András Péter, http://solomoncoder.com), which in turn was mapped to the inertial data gathered. From each continuous segment of tagged data we clipped a second from both ends to reduce possible coder error and segmented the data using a window of 1.3 seconds and steps of 0.5 seconds. From each of the segments thus coded we calculated the corresponding vector in the feature space. For the detailed description of the feature space see S3 Table.

#### Models and parameters

Again following the method described in [25] Support Vector Machine (SVM) models were used to test the different strategies introduced in the next section. As an implementation, svm.SVC from the python module scikit-learn version 0.22.1, with python version 3.7.5 was used. The SVM model was initialized with the rbf (Radial Basis Function) kernel, which has two parameters, *C* and *γ*. This implementation uses the “one-versus-one” approach for multi-class classification, and can provide class probability estimates based on [37].

When optimizing for a specific measure of performance, a grid search in the {*C*, *γ*} parameterspace was ran, carried out on the Atlasz HPC of the Eötvös Loránd University. The grid points were evenly spaced on a logarithmic scale, with 27 points between 10^−5^ and 10^9^ for *C* and 24 points between 10^−9^ and 10^3^ for *γ*, totalling 648 points. The parameter pair with the highest measure of performance was chosen for models in subsequent analyses.

Data and code can be found at [38].

### Measuring model performance

Machine learning models are trained on one part of the data (training set) and the performance measured on the other part of the data (test set). Thus a performance metric and a data splitting strategy needs to be chosen. During the evaluation of behaviour classification models, we must keep in mind, that the ultimate goal of such models is to transform the raw data into a sequence of behaviours the ethologist can analyse from an ethological viewpoint.

#### Measures of accuracy

Although other metrics exist, such as precision, recall and the F-measure (see e.g. [39]), we consider two ways to measure accuracy as these are the most common metrics employed studies of animal behaviour recognition.

The general definition of accuracy for a binary classificator is the following [40]:

$$\begin{array}{c} accuracy=\frac{TP+TN}{TP+TN+FP+FN} \end{array}$$ (1)

where *TP* stands for *true positive*, *TN* for *true negative*, *FP* for *false positive* and *FN* for *false negative*. In the binary case the definition of these is straightforward. For example, we could ask our model whether the dog is running at a given time or not. If the dog *is* running and the model answers *run* then we have a *true positive*, if the answer is *not run* we have a *false negative*. Conversely, if the dog is actually *not* running and the model answers *run* we have a *false positive* and if it answers *not run* we have a *true negative*. The generalization of the above to non-binary classificators is a non-trivial problem, but since animal behaviour is inherently non-binary, both metrics used in the field offer a solution to the problem.

- **Overall accuracy**: This is likely the most straightforward generalization. For a given segment of data the machine learning model returns a probability for each possible behaviour, and the one with the highest probability is taken as the model’s answer. To get *overall accuracy* the number of segments are counted where the model correctly guessed the true class, which is then divided by the total number of segments.
- **Threshold accuracy**: This measure uses the probability returned by the classifier [41] in a more complex manner. The method defines a probability threshold: a correct identification becomes a *true positive* if the associated probability is above the threshold and a *false negative* if below the threshold. Conversely, an incorrect identification becomes a *false positive* if the associated probability is above the threshold and a *true negative* if below the threshold. After categorizing the response to all segments in this manner Eq 1 is applied.

When optimizing for *threshold accuracy*, one must also optimize for the probability threshold. In the subsequent analyses, when optimizing for *threshold accuracy*, the metric was calculated with a probability threshold of 0.5, 0.6, 0.7, 0.8 and 0.9 and the one that resulted in the highest value of the metric was chosen.

From the ethologists’ perspective, there is big difference in how the two methods label behaviour segments. When using *overall accuracy* all of the segments of the data are labelled with a behaviour, while using *threshold accuracy* segments where the probability falls below the threshold are essentially unlabelled. Thus a high threshold, could lead to only a few labels being assigned, albeit with very high probability, which may be undesirable.

#### Cross-validation strategies

In this article two cross-validation method are explored, the commonly used *random* method [42], and the proposed *per individual* method, which we argue is better suited in case of the typical use case of animal behavioural classification (observation of otherwise unobservable animals):

- **Random method**: The entire dataset is segmented randomly, typically with 70% of data used for training the model and 30% of the data used for testing the model. In an *n-fold cross-validation* setup this is repeated *n* times (with the suggested number of folds being 5-10 [43]), and the average accuracy of these is reported as the accuracy. In this case a single individual is likely to contribute data to both the training and the testing.
- **Per individual method**: Data is segmented per individual, so that the entire data of one individual is taken as the test data and the rest of the data is used to train the model (thus this is not the leave-one-out method advised against, since in that method single datapoints are left out [43]). This is repeated for all animals and the average accuracy of these is reported as the accuracy. In this case an individual never contributes to both the testing and the training, which is closer to some use-case scenarios, where previously unrecorded individuals’ data is classified by the model.

When using the per individual method, since each of the animal groups consisted of 7 animals, we perform 7 calculations. To make the random method comparable, we used a 7-fold cross-validation in those cases.

## Results and discussion

### Comparison of accuracy metrics

**Fig 2:**
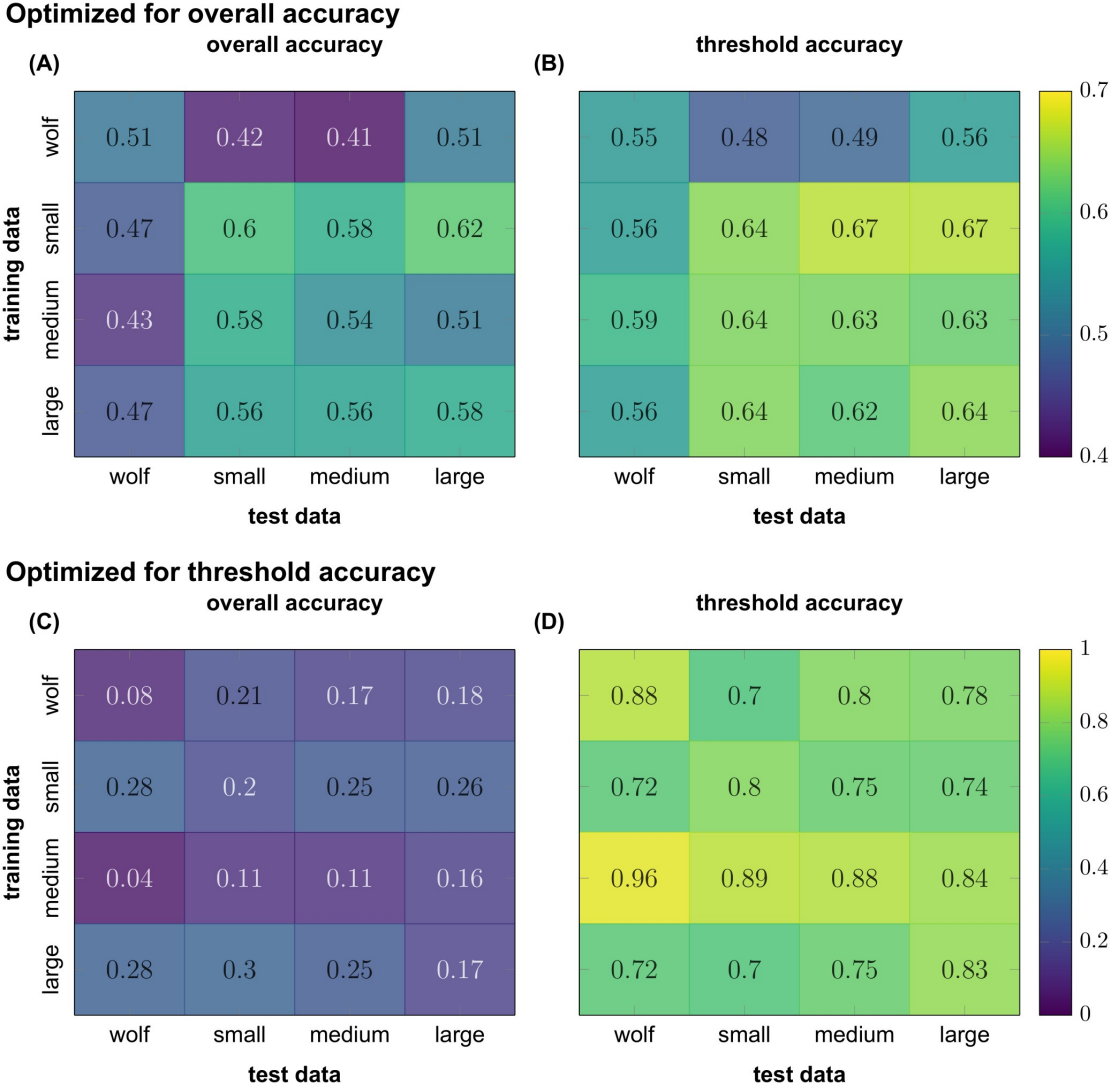
Performance of our models, under different optimization strategies. Small, medium and large are three groups of dogs, with differing sized dogs in them. Panels (A) and (B) show overall and threshold accuracies of models optimized for overall accuracies, while panels (C) and (D) show the same for models optimized for threshold accuracies. Optimizing for threshold accuracy leads to unbalanced models, with lot of true negatives, but few true positives.

First we trained four models on the data of the four groups of animals (small, medium and large dogs, and wolves). Parameters were optimized to maximize overall accuracy and the metric was measured with the per individual method (see Cross-validation strategies). In Fig 2 panel (A) we show the cross-validated overall accuracies for each group in the diagonal. Each optimized model is then used to predict the data of the other groups, for which the overall accuracies are also shown on Fig 2. On panel B we show the same models, but instead of the overall accuracies the calculated threshold accuracies. Although the overall accuracy and the threshold accuracy naturally differ, in this case they are quite similar and all well above the theoretical chance level of 0.125 (see Measures of accuracy for the definitions of accuracies).

In contrast, we show the same construct on Fig 2 panel (C) and (D), but this time with the parameters optimized to maximize the threshold accuracy using per individual cross-validation for each group. In this case the overall accuracy of the cross-validations in two groups (wolves and medium dogs) dropped below the theoretical chance level of 0.125, but all overall accuracies are generally very low compared to the case of optimizing for overall accuracy, while threshold accuracies became very high compared to both current and previous overall accuracies and the previous threshold accuracies as well.

Our data suggests, that optimizing for threshold accuracy leads to a less balanced model that favours discarding data, rather than making a less well established guess about the behaviour. We argue that the latter is more suitable for ethologists monitoring the behaviour of animal as it will provide a less precise, but more even representation of an animal’s long term behaviour.

### Comparison of cross-validation strategies

**Fig 3:**
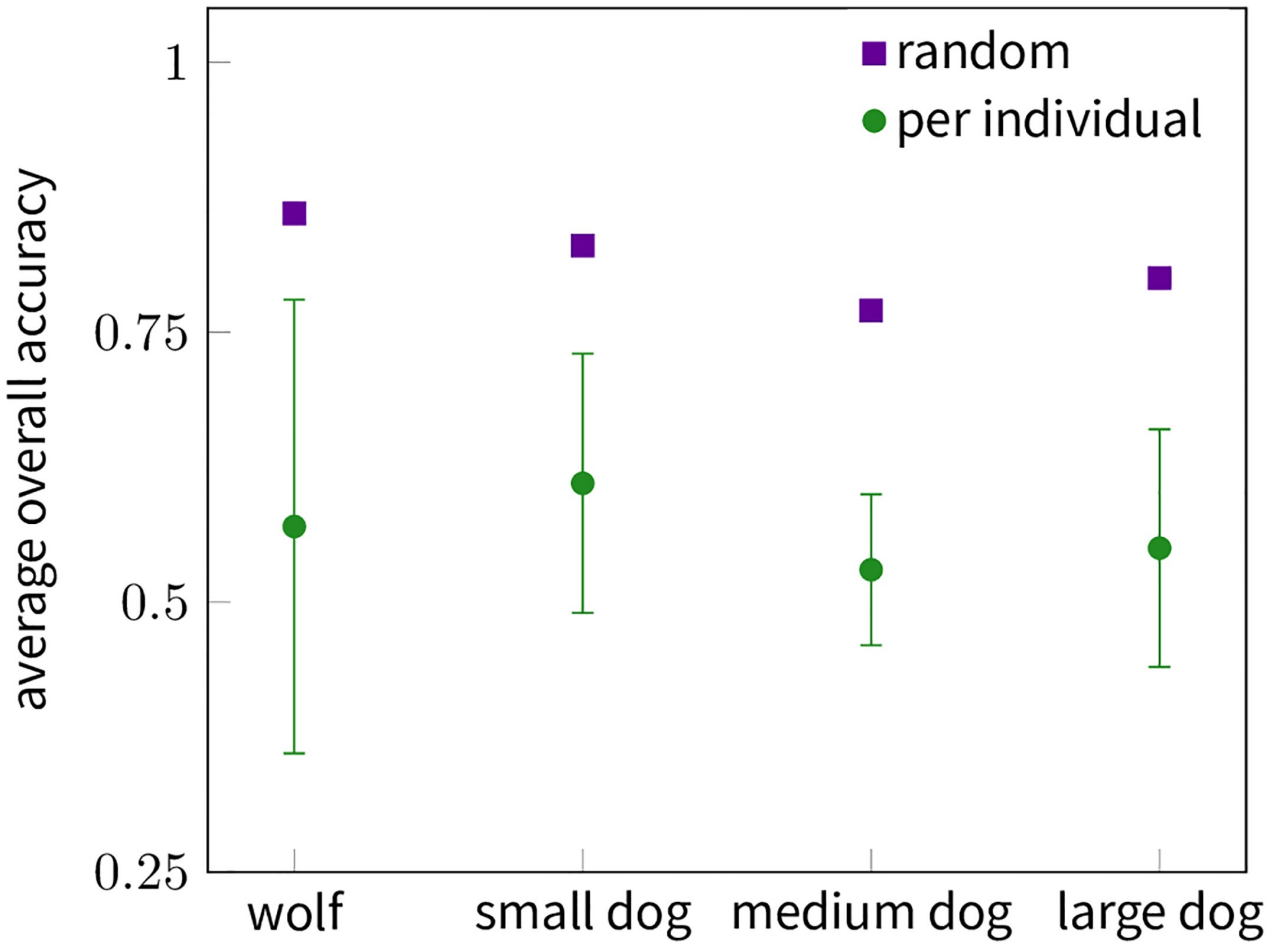
Overall accuracy of cross-validations with different strategies. In the “per individual” case, overall accuracies were calculated by using one animal as the test data and the other six animals to build the model. The figure shows the average of these 7 evaluation with a 95% confidence interval. In the “random” case 30% of the data was chosen randomly to serve as test data and the rest was used to build the model and the overall accuracy calculated. For fair comparison the figure presents the average of 7 such randomized tests (the confidence intervals are smaller than the markers). Due to the fact that in the randomized case a single animal is highly likely to contribute both to the training and the test data, much higher accuracies are reached, but using “per individual” gives a better estimate of the model’s performance when applied to specimens without labelled data.

A related issue arises, when we consider the above described use case of behavioural classification. A very common method used for cross-validation is randomly splitting data between a training set and a test set (see Cross-validation strategies). The rationale behind splitting the data is that having the same data point in the training set as in the testing set would bias the performance metric, making it seem to perform better than it would in the general case. This works well, when the dataset has uncorrelated data points or the correlation is minimal, but this assumption typically does not stand for behavioural data. Due to methodological issues typical datasets are created from a handful of animals, while each animal contributes hundreds or thousands of data points, thus the data is highly correlated. Since a very typical goal of training behavioural classificator models is to be able to use them to observe animals from which we do not have any labelled data [35, 36], in this case we should be interested in how well our model performs on novel animals rather than how it performs on the already observed animals. Our suggested method of doing a per individual cross-validation (see Cross-validation strategies) is a better proxy for this, since the training data never includes data from the animal we use for assessing performance. On Fig 3 we present a comparisons of the two cross-validation strategies for each of our animal groups, clearly showing that the random method gives much higher estimates of overall accuracy. We suggest that the performance of models trained on the data of few animals and assessed with the random cross-validation strategy—as is the most common case in the field—are highly overestimated.

### Cross-species performance of models

A combined dog model was built which was optimized using cross-validation without including wolf data, and thus was not optimized for performing well on wolf data. We note, that nevertheless, when data from both species is available (although one of them far exceeding the other), combining both species’ data might yield better results, but this was not investigated.

On Fig 2 it was already shown that the models built from the various dog groups perform 4 to 8% worse on the wolf data, than the wolves’ own cross-validation. The physical similarity in size does not correlate with the accuracy, which is line with findings that gait similarities between dog breeds are influenced by more factors than weight and relative velocity [44]. To increase accuracy, a model was trained using the data of the combined dog groups, with which a 49% overall accuracy was reached (only 2% less than the wolves own cross-validation).

**Fig 4:**
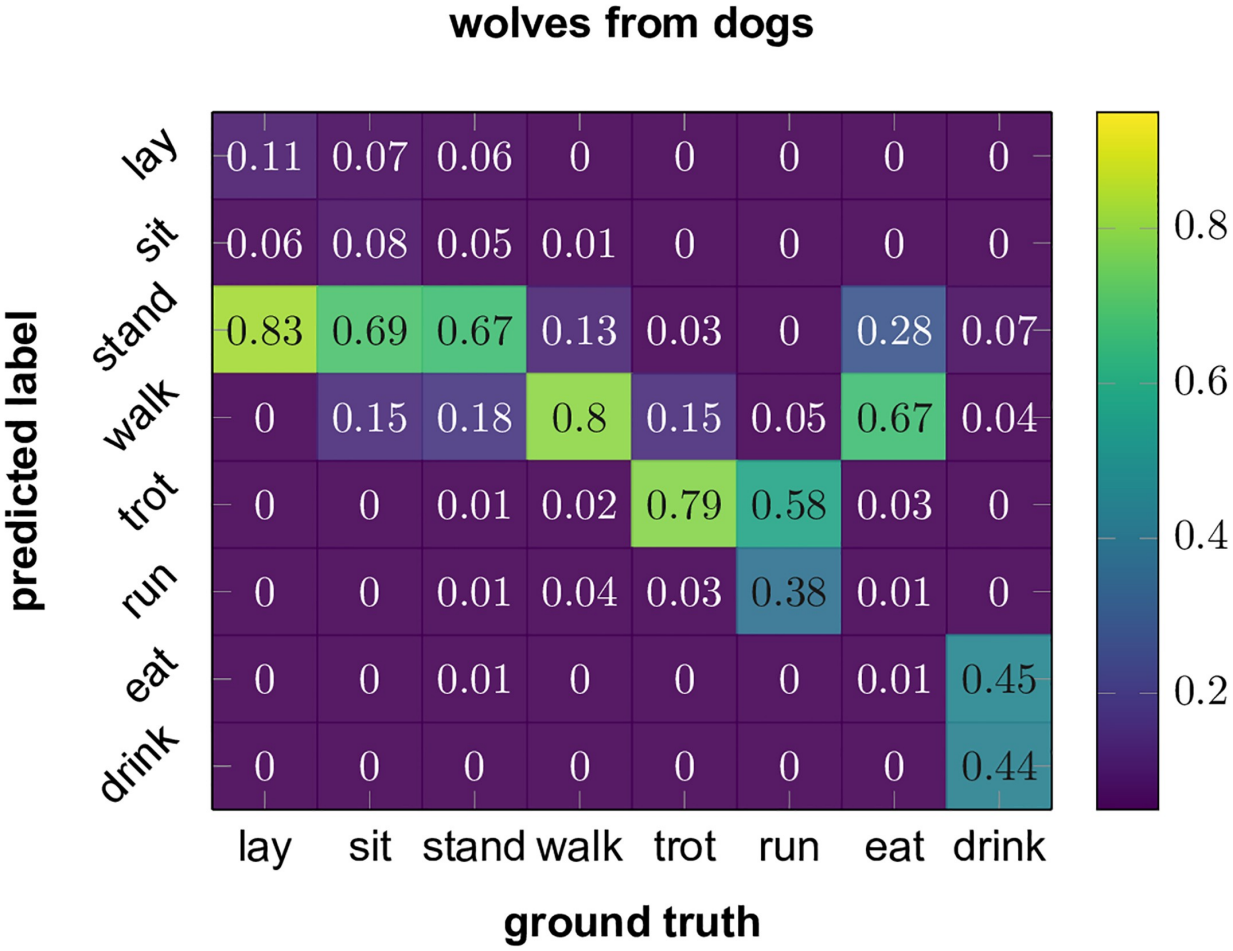
Confusion matrix between dogs and wolves. Using the combined data of dogs to predict wolves. Differentiating between the passive states (sit, lay, stand) is problematic, but they can be combined with great accuracy. Walk and trot are easily distinguishable, while trot and run are often mixed up, the data suggest wolves may in general run faster than dogs. Feeding behaviour seems to be the most problematic, although the drinking of wolves is practically always considered as feeding behaviour.

To gain a more detailed insight to how well cross-species models perform we present the confusion matrix of the combined dog model used to predict the wolf data on Fig 4. In general, we can see, that differentiating the three non-moving behaviours (lay, sit stand) is a non-trivial task, although regarding them as a combined “passive” behaviour, the accuracy is much higher. The three locomotion modes are ordered in the figures by the speed of the motion (walk, trot, run). We see that the error is always in the direction of classifying the movement as slower, but this only becomes a very serious issue for categorizing “runs” as “trots”. This might be due to the fact that in general dogs were more enthusiastic in performing the tasks.

Feeding behaviour seems to be the most problematic. Drinking is 99% of the time classified as a feeding behaviour (drinking or eating), but eating is in practice always misclassified. We believe this is due to a methodological error on our part. In case of drinking the way water was presented to the animals are inherently not very different for both species, since they both drank from a more-or-less ground level pool of water (actual pool for wolves and drinking bowl for dogs). In contrast, dogs were fed canned or dry dog food from feeding bowls, while wolves were presented with large pieces of raw meat on the ground, as is part of their natural diet. This is a lesson, that in these cases care must be taken to collect behaviour in scenarios that would happen in the wild, outside of the anthropogenic world. Although only short behaviour elements are needed to be collected (which are unlikely to differ between captive and free-ranging conspecifics), this could become problematic, if some behaviours can not be elicited in captivity at all.

All-in-all, we believe, that with more careful data collection, it is feasible to monitor the behaviour of wolves, including feeding behaviour, with models trained on dog data.

Results could be improved in numerous ways. The data loggers used were attached to a collar, that can move around depending on how much fur the animal has. Borrowing techniques developed for mobile phone with changing orientation could improve differentiating between the passive behaviours [19]. Increasing the amount of available data by including other sensors in the collar (e.g. GPS, microphone) could also help. A more complicated improvement could be placing several devices on the animals body, e.g. adding a harness to separate head and body movement, or adding a small accelerometer to each leg, although such a setup might be hard to carry out in practice with wild animals.

The practicing ethologist can use the above method to label large amounts of behaviour data, that would not be possible with only human experts. The increased amount of data can mitigate the effect of random errors, however new sources of errors are introduced. For labels, where the accuracy is very high, traditional methods can be used, thus efforts should be mainly aimed at increasing the accuracy of such models. Lower accuracy results can also be used however, if instead of using the most probable output as the behaviour, one treats the output of the classification as inherently probabilistic ensemble, and modifies the subsequent statistical analyses to accommodate this.

## Conclusions

We found, that for the use case of a typical ethological study, using overall accuracy with per individual cross-validation is preferable. We show that cross-species classification of behaviour is feasible between closely related species.

Our results highlight the need to create a standardized set of best practices in evaluating and reporting results of behaviour classification models. This would not only be important for increased comparability between studies, but also for the future ethologist choosing between automated behaviour classification tools.

In this paper only a limited set of practices were examined, thus more research is needed to establish such a list of best practices. Applying machine learning models to data is the last step in the process, so research must also extend to previous steps, including, but not limited to the creation of ethograms, data labelling practices, or the types of metadata that needs to be reported.

## Supporting information

S1 Table Basic data about the subjects used in the experiment, as indicated by owners. (DOCX)

S2 Table Definitions of the behaviour categories. (DOCX)

S3 Table The components of the featurespace. (DOCX)
