# Supplementary material for: Challenges of machine learning model validation using correlated behaviour data: Evaluation of cross-validation strategies and accuracy measures
Source: PLoS One. 2020 Jul 20;15(7):e0236092. doi: 10.1371/journal.pone.0236092 (PMC7371169; doi:10.1371/journal.pone.0236092)
Supplement: S1 Table — (DOCX) [file pone.0236092.s001.docx]

| **#** | **Breed/Species** | **Weight (kg)** | **Body size** | **Age**  **(years)** | **Sex** |
| --- | --- | --- | --- | --- | --- |
| 1 | Miniature Schnautzer | 7 | small | 2 | Neutered female |
| 2 | West Highland White terrier | 7 | small | 8 | Neutered female |
| 3 | Whippet | 8 | small | 11 | Neutered female |
| 4 | Mixed breed | 6 | small | 1.5 | Neutered male |
| 5 | Jack Russell terrier | 6.5 | small | 2.5 | Neutered male |
| 6 | West Highland White terrier | 9 | small | 5 | Male |
| 7 | Cairn terrier | 10 | small | 7 | Neutered male |
| 8 | Samoyed | 20 | medium | 1.3 | Female |
| 9 | Border Collie | 14 | medium | 12 | Female |
| 10 | Mudi | 16 | medium | 9 | Male |
| 11 | Mixed breed | 23 | medium | 4 | Neutered female |
| 12 | Mixed breed | 12 | medium | 5 | Neutered male |
| 13 | Beagle | 17 | medium | 6 | Neutered male |
| 14 | Mixed breed | 16 | medium | 10 | Neutered female |
| 15 | Mixed breed | 30 | large | 8 | Neutered male |
| 16 | Hungarian Vizsla | 28 | large | 3 | Male |
| 17 | Mixed breed | 40 | large | 4 | Neutered male |
| 18 | Husky | 35 | large | 5 | Male |
| 19 | Husky | 32 | large | 7 | Female |
| 20 | Mixed breed | 25 | large | 1.5 | Neutered male |
| 21 | German Shorthaired Pointer | 40 | large | 11 | Female |
| 22 | Mixed breed | 30 | large | 8 | Neutered male |
| 23 | Grey wolf | 35 | large | 5 | Female |
| 24 | Grey wolf | 35 | large | 4 | Male |
| 25 | Grey wolf | 30 | large | 1.5 | Female |
| 26 | Grey wolf | 30 | large | 1.5 | Male |
| 27 | Grey wolf | 30 | large | 1.5 | Male |
| 28 | Grey wolf | 30 | large | 1.5 | Male |
| 29 | Grey wolf | 30 | large | 1.5 | Male |

**Table S1: Basic data about the subjects used in the experiment, as indicated by owners**
