# Supplementary material for: Challenges of machine learning model validation using correlated behaviour data: Evaluation of cross-validation strategies and accuracy measures
Source: PLoS One. 2020 Jul 20;15(7):e0236092. doi: 10.1371/journal.pone.0236092 (PMC7371169; doi:10.1371/journal.pone.0236092)
Supplement: S2 Table — (DOCX) [file pone.0236092.s002.docx]

| **Name** | **Definition of the behavioural category** |
| --- | --- |
| **Stand** | The animal stays at one place with four straight legs touching the ground. Movements of the tail and the head are allowed for. |
| **Sit** | The animal stays at one place with front legs in extended position, both hind legs bent and the metatarsal regions touch the ground. Movements of the tail and the head are allowed for. |
| **Lay** | The animal stays at one place in sternal recumbency with all four legs bent and both elbows touching the ground. Movements of the tail and the head are allowed for. |
| **Walk** | The animal is using the slowest, symmetric four-beat gait, by which it supports its weight with its feet in the following sequence: front left limb, hind right limb, front right limb, hind right limb [1]. |
| **Trot** | The animal is performing a symmetric gait by which the diagonal pairs of limbs move almost simultaneously [2]. |
| **Run** | The animal is performing an asymmetrical, three- (i.e. canter) or four-beat (i.e. gallop) gait. During canter, the gait pattern is a hind foot, the opposite hind foot and its front diagonal, followed by the other front foot and suspension, when present [2]. In gallop the animal supports its weight with its feet in the following sequence: right front leg, left front leg, right hind leg, left hind leg. Just after taking off from the front left foot the dog achieves suspension [2]. |

**Table S1: Definitions of the behaviour categories**

**References**

[1] Coros S, Karpathy A, Jones B, Reveret L, Van De Panne M (2011) Locomotion skills for simulated quadrupeds. ACM Transactions on Graphics (TOG) 30: 59.

[2] Nunamaker D, Blauner P (1985) Normal and abnormal gait. Textbook of small animal orthopaedics International veterinary information service, USA.
