# Supplementary material for: Challenges of machine learning model validation using correlated behaviour data: Evaluation of cross-validation strategies and accuracy measures
Source: PLoS One. 2020 Jul 20;15(7):e0236092. doi: 10.1371/journal.pone.0236092 (PMC7371169; doi:10.1371/journal.pone.0236092)
Supplement: S3 Table — (DOCX) [file pone.0236092.s003.docx]

| **Base parameter source** | **Base parameter components** | **Comments** |
| --- | --- | --- |
| Gravitational acceleration (*g)* | *g*_x_, *g*_y_, *g*_z_ | The acceleration of the device is the sum of the acting gravitational acceleration and the acceleration induced by the movement of the animal. The API of the data logger allows access to the gravitational component. |
| User acceleration (*a*) | *a*_x_, *a*_y_, *a*_z_ | This is the gravitation acceleration subtracted from the total acceleration. |
| Angular velocity (*ω*) | *ω*_x_, *ω*_y_, *ω*_z_ | The angular velocity of the device. |
| Derivatives of the above | 9 quantities | Numerical derivatives. |
| Dot products | ***a·g***, ***a·ω***, ***ω·g*** |  |
| **Components of the featurespace** | **Definition (for any base parameter component *x*)** | **Comments** |
| 1st, 2nd and 3rd moments | *μ* = E[*x*] = ∑*x*/*n* *σ* = (E[(*x*–*μ*)^2^])^1/2^  *γ* = E[((*x*–*μ*)/*σ*)^3^] | i.e., average (mean), standard deviation, skewness |
| Extrema values | min(*x*), max(*x*), ext_count(*x*) | Ext_count is the total number of local minima and maxima. |
| Fast Fourier Transformation components | DC and the power of the three largest components of the spectrum and the corresponding frequencies. | Largest as in, has the highest power. They are saved in order of DC component, then highest to lowest, with power and frequency saved alternatingly. |

**Table S3: The components of the featurespace.**

There are 21 base components calculated, each being a sequence which together correspond to a segment of the data. The components of the featurespace are made up of the listed components sequentially calculated on each base component in the order listed, making the featurespace 21*12
